# Health consequences of graded, full, and no sickness absence among workers with musculoskeletal disorders: health profiles and six-months symptom changes of patients referred to Norwegian outpatient clinics for chronic neck and back pain

**DOI:** 10.1186/s12891-025-08570-7

**Published:** 2025-05-01

**Authors:** Samineh Sanatkar, Sharon A. M. Stevelink, Nils Aars, Ingvild Bardal, Oda Lekve Brandseth, Beate Brinchmann, Arnstein Mykletun

**Affiliations:** 1 Black Dog Institute, School of Psychiatry, UNSW Sydney, Hospital Road, Randwick, NSW 2031 Australia; 2 Centre for Work and Mental Health, Nordland Hospital Trust, Bodø, Norway; 3 Department of Psychological Medicine, Institute of Psychiatry, Psychology and Neuroscience, King’s College London, London, UK; 4 King’s Centre for Military Health Research, Department of Psychological Medicine, Institute of Psychiatry, Psychology and Neuroscience, King’s College London, London, UK; 5 Centre for Research and Education in Forensic Psychiatry, Haukeland University Hospital, Bergen, Norway; 6 Department of Community Medicine, UiT- The Arctic University of Norway, Tromsø, Norway; 7 Division for Health Services, Norwegian Institute of Public Health, Oslo, Norway

**Keywords:** Health service utilisation, Sickness absence, Musculoskeletal disorder, Neck and back pain, Quality of life

## Abstract

**Objective:**

It is generally assumed that graded sickness absence results in favourable health effects due to observed positive consequences of maintaining work participation. To date, however, the direct health benefits of graded sick leave have not been widely explored. Musculoskeletal disorders are among the most prominent health issues resulting in work incapacities. This study examined baseline characteristics and six-months pain-related disability and health-related life quality progression of working age adults who attended a neck and back pain outpatient clinic. Patients prescribed graded sick leave were compared to patients prescribed full sick leave and those working without sick leave.

**Methods:**

Demographic, health, and treatment information of patients were assessed using clinician and patient self-report questionnaire data collected at neck and back pain outpatient clinics between 2016 and 2022. Data were obtained from the Norwegian Neck and Back Registry and the Norwegian Labour and Welfare Administration. Patient characteristics in the two weeks leading up to clinic intake were described. General linear models for repeated measures were employed to observe six-months changes in pain-related disability and health-related life quality.

**Results:**

A total of 5143 (54% female, *M* = 44.70 years, *SD* = 11.50) patients were prescribed full (*n* = 1411, 27%), graded (*n* = 1164, 23%), and no (*n* = 2568, 50%) sickness absence. Patients prescribed graded sick leave reported lower baseline levels of pain-related disability compared to those on full sick leave but higher pain-related disability than patients without sick leave. There were significant main and interaction effects of time and sickness absence, whereby reductions in pain-related disability were greatest among patients prescribed full sick leave, however, this group reported the highest levels of pain-related disability and lowest life quality prior to their clinic intake and six months later.

**Conclusion:**

Patients who were prescribed full, graded, or no sick leave exhibited significant, albeit not clinically meaningful, reductions in pain-related disability over a six-months period. Symptom reductions may be due to clinician support or remission trends in line with regression towards the mean. While no superior health effects of graded sick leave were noted, work participation did not appear to have detrimental health effects.

**Supplementary Information:**

The online version contains supplementary material available at 10.1186/s12891-025-08570-7.

## Background

Scandinavian countries emphasise benefits of social welfare and provide health care, rehabilitation, and income support through various sickness and disability benefit schemes to their citizens. Norway provides the most comprehensive support to persons on sick leave compared to other OECD-countries [1], including full wage compensation for one year within a 1.5-year period. Despite high participation in the Norwegian labour force, the economic costs of work absenteeism are particularly pronounced in this country, amounting to about 5% of Norway’s gross domestic product [2]. To address this problem, a target was set by government, industry and labour unions to measurably reduce sickness absence rates [3]. To this day, however, Norwegian sickness absence rates have remained fairly stable over the last decade [4].

Musculoskeletal disorders are one of the main contributors to sickness absence in Norway [5]. These disorders describe pain and injury of the locomotor system. Globally, musculoskeletal disorders make up about two thirds of the estimated rehabilitation needs of working adults and are the main contributors to years lived with disability [6]. As a consequence, musculoskeletal disorders are the most common causes of work incapacity in Norway [7], with over one fourth of Norwegian long-term sickness absence certifications of six months or more attributed to musculoskeletal issues [8, 9].

While unsafe and distressing work conditions have been shown to exert detrimental effects [10], work participation has generally been proposed as a protective factor to promote functional recovery and prevent long-term illness. Newly published recommendations explicitly list work participation to help increase health and quality of life outcomes in people diagnosed with rheumatic and musculoskeletal disorders [11]. This can be explained by the nature of musculoskeletal disorders, by which some activity may be healthy and aid functional recovery [12]. Given the large proportion of musculoskeletal disorders contributing to national sickness absence rates and considering the increased mortality risk associated with disability [13, 14], investigating the potential health and quality of life benefits of this approach is warranted. Where full-time work commitments cannot be maintained when diagnosed with musculoskeletal disorders, part-time or graded sick leave has been investigated as a superior method for achieving return to work outcomes compared to full sick leave [15, 16]. During part-time or graded sick leave, sick listed employees take on work duties on a reduced schedule until health and functioning is sufficiently restored to return to full work duties. Part-time or graded sick leave may also be superior to staying in the workforce without sick leave, as taking no time off can delay preventive care and medical treatment [17, 18].

To date, the majority of studies examining graded sick leave have investigated work recovery outcomes but did not explicitly measure whether health symptoms improved accordingly, with notable exceptions examined in Norway, the Netherlands, and Finland [19–21]. For example, Standal and colleagues (2021) found that graded sick leave was most common in Norwegian workers who reported medium levels of self-reported health. The authors argued that, if workers felt healthy, it was more likely that they skipped graded sick leave altogether and returned to work in full capacity. Conversely, if workers considered themselves to be in very poor health, they would be too ill to work [20]. This finding suggests that self-reported health status may constitute a useful, easy-to-measure indicator of whether graded sick leave should be considered. However, complicating this picture, Canadian research evidence suggests that the advantages of partial return to work can materialise no sooner than six months for workers with severe musculoskeletal disorders [22]. This highlights the importance of measuring the progression of self-reported health status alongside return to work outcomes for workers with musculoskeletal disorders.

Considering the above, it is important to examine the influence of work participation on health improvement, especially among workers with musculoskeletal disorders. This is because many workers with musculoskeletal disorders report working despite being restricted in carrying out daily activities [23]. It is possible that the continuation of work commitments delays functional recovery, especially when work risk factors are present, which may outweigh the positive effects of work [24]. In other words, while return-to-work outcomes can be seen as a proxy for functional recovery after illness, improvement of pain-related disability and a return to good self-perceived quality of life would constitute direct, and we argue important, measures of health progression under any sickness absence scheme.

With this exploratory study, we observed patients with musculoskeletal disorders depending on their sick leave status. First, we compared musculoskeletal patient characteristics of (1) employees who followed a graded sickness absence program combining sick leave with part-time work, (2) employees who were on full sick leave, and (3) employees who worked without sick leave. Second, we examined whether the three patient groups differed with regards to mean changes in pain-related disability and health-related quality of life across two time points, between the baseline patient questionnaire and a 6-months follow-up questionnaire.

## Methods

### Design and sample

Questionnaire data from 2016 to 2022 was obtained through the Norwegian Neck and Back Register (Norsk nakke- og ryggregister; NNRR), a national medical quality register established to monitor outcomes from patients attending neck and back pain outpatient clinics in the specialist health sector^1^. To date, there are 12 such outpatient clinics located within the South-East, Western, Central, and Northern Regional Health Authorities that have contributed data to the registry. All 12 outpatient clinics provide multidisciplinary specialist healthcare services and are connected to their hospital’s Department of Physical Medicine and Rehabilitation. Clinicians at outpatient clinics are medical doctors and physiotherapists who provide health care consisting of assessment and treatment (including pharmacological, physical rehabilitation, and pain reduction therapies) with follow-up consultations. Patients are referred to an outpatient clinic by their general practitioner, consultant physician, manual therapist, or psychologist. Referral considerations are made in line with treatment prescriptions outlined in national guidelines [25], which are similar to international standards [26] that call for multidisciplinary approaches if patients’ symptom severity, duration, and recurrence warrant extended treatment. After referral, wait times for assessments at outpatient clinics range between 12 and 26 weeks.

NNRR staff oversee the distribution and collection of a standard set of online questionnaires through a digital health platform, which links into the 12 outpatient clinics’ digital infrastructure. In the two weeks prior to their first appointment, patients receive an online baseline questionnaire through the digital health platform that comprises of demographic information, treatment history, medication, mental and physical health, and employment. During the examination, the patient’s clinician is required to record diagnosis and treatment information in a clinician survey, which is captured by the NNRR. The number of appointments at the outpatient clinic is generally restricted to one or two contacts irrespective of the severity of a patient’s condition, however, patients with more severe symptoms may be referred to other hospital departments for further treatment. Patient follow-up questionnaires take place at 6 months and, since 2022, at 12 months following the initial appointment and are also stored in the NNRR. Data derived from NNRR has been utilised in recent research articles [27–29]. This study utilised clinician responses relating to diagnosis and patient responses at intake and 6 months. Baseline patient questionnaire items can be accessed through the NNRR website [30].

Sick leave and work status information was derived from the Norwegian Labour and Welfare Administration (Arbeids- og velferdsetaten; NAV). NAV coordinates and administers the national benefit and pension schemes, including unemployment and sickness benefits, work assessment allowances and pensions. In this study, we linked patients who took part in the NNRR questionnaire surveys to the NAV register through their national personal identifier to obtain reliable work and sick leave information.

**Fig. 1:**
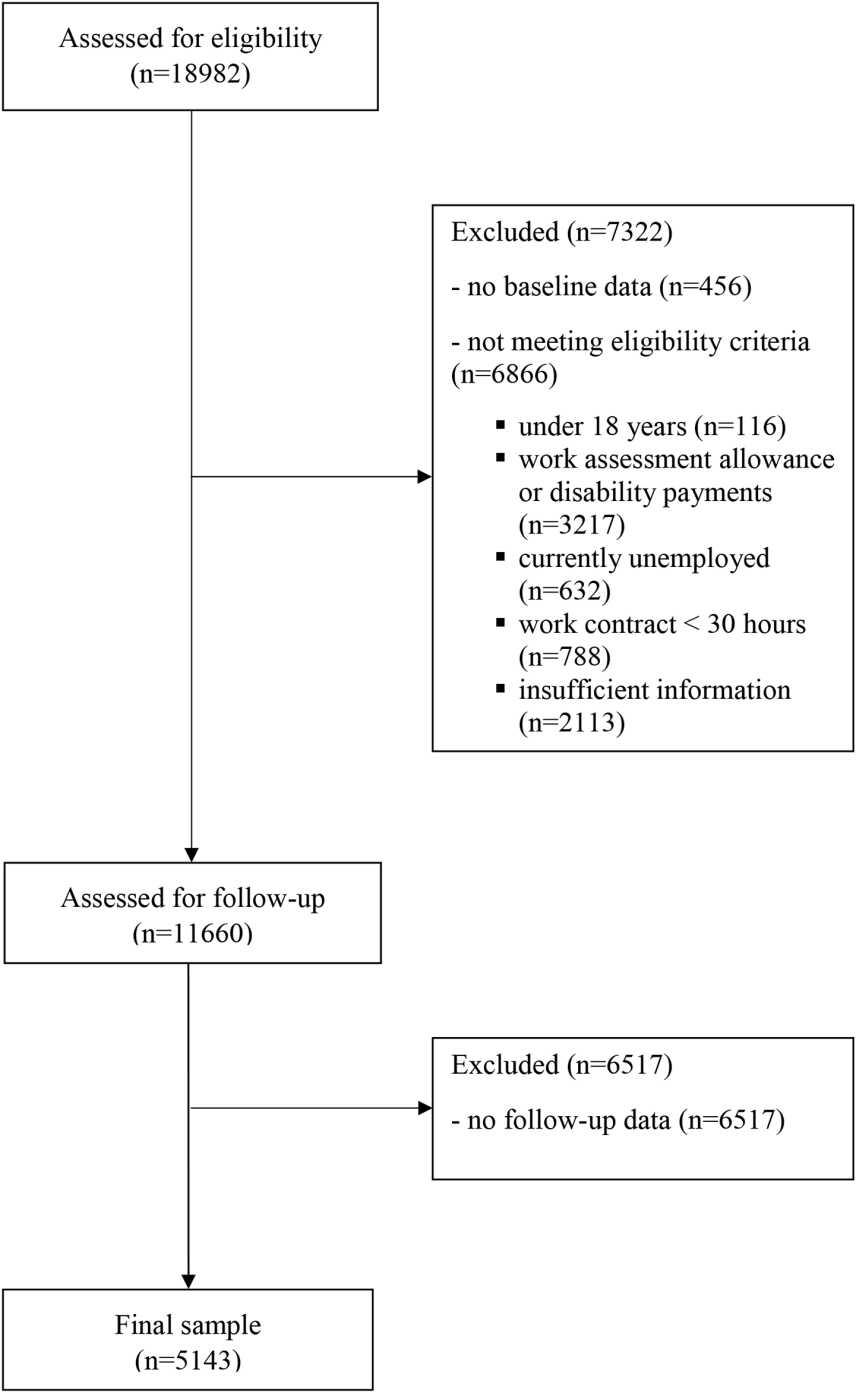
Flow diagram illustrating the patient selection process

A flow diagram illustrating the full selection process is presented in Fig. 1. After removal of duplicate patient identifiers, a total of 18,526 patients returned the baseline questionnaire prior to their initial appointment, of which 8238 (44.47%) also returned the 6-months follow-up questionnaire. No a priori power calculation was conducted. Instead, a selection of patients who met employment, age, and questionnaire completion criteria were included in the present analysis. Patients who were not employed (*n* = 632, 3.41%) or were recipients of long-term rehabilitation benefits (i.e., work assessment allowance and disability payments issued by NAV, *n* = 3217, 17.36%) at the time of the baseline questionnaire were excluded from analysis, as were patients under 18 years of age (*n* = 116, 0.63%). To ease interpretation of work participation according to categories of sickness absence, we further excluded employees who were listed with NAV as contractually working less than 30 h per week (*n* = 788, 4.25%). We made this selection to ensure that sickness absence and no sickness absence groups differed in expected ways in their degree of work participation. Taking into account incomplete records (*n* = 2113, 11.4%) and missing follow-up questionnaires of eligible patients (*n* = 6517, 35.18%), this resulted in a final sample of 5143 (27.76%).

### Ethics approval and consent to participate

Patients provided informed consent for data collection and storage in the NNRR and consented to the use of their responses for research purposes and linkage to other registry data. Approval to obtain data from outpatient clinics was sought from the NNRR council and confirmed on April 7th, 2022. An application to obtain NAV welfare data was approved on January 24th, 2023 (approval number #22/18149). The Regional Ethics Committee North approved this project under the protocol number #138597.

## Measures

### Sociodemographic characteristics

The baseline questionnaire given to patients prior to their intake appointment collected a number of sociodemographic, health, and mental health information. Demographic items collected were, amongst others, patient age, sex, education level and occupation. Self-reported information concerned workplace characteristics (e.g., current work ability, job satisfaction, and positive employer beliefs around whether the patient believes their employer would like them to return to work) and pain experiences (e.g., duration of current pain, number of painful body regions, and pain medication usage). Fear of physical and work activity was assessed using the Fear-Avoidance Beliefs Questionnaire [31] developed for back pain patients. Patients were further asked to complete the 10-item short form of the Hopkins Symptom Checklist [32], which measures mental health symptoms of anxiety and depression. Diagnostic information was taken from the clinician version of the intake questionnaire.

### Sickness absence status

In Norway, sickness absence is prescribed by the treating practitioner (usually a patient’s general practitioner). Employees are entitled to benefits from the first day of their prescribed sick leave. The employer liability period, in which employers continue to pay their employees’ usual salary, is 16 days. Subsequently, NAV determines payouts of sickness benefits that provide income support. Sickness absence prescriptions are captured and dated in NAV records. NAV employment status and sick leave information were used to determine sickness absence status of patients at the time of the baseline questionnaire. Patients were categorised into one of three categories, (1) employed without sickness absence (i.e., 0% sick leave), (2) employed with graded sickness absence (i.e., between 20 and 95% sick leave as regulated by NAV), and (3) employed with full sickness absence (i.e., 100% sick leave).^2^

### Low back pain-related disability

Patients completed the 10-item Oswestry Disability Index Version 2 [33, 34] (ODI) to assess low back pain-related disabilityat the first neck and back outpatient clinic assessment and 6 months later. ODI measures back and leg pain severity as well as functional impairment in areas of everyday life (e.g., personal care, walking, sleeping) as a consequence of the pain experience. Respondents indicated their answers on a 6-point Likert-type scale ranging from 0 to 5, whereby higher values indicated more severe pain and impairment. To create index scores, response values were summed and expressed as a percentage. Consequently, ODI scores could range from 0 to 100%, with functional limitation categories of 0–20% = Minimal, 21–40% = Moderate, 41–60% = Severe, 61–80% = Very severe, and 81–100% = Bedridden or overreported [33]. Internal consistency of ODI items was high, with a Cronbach’s alpha value of 0.85, comparable with other studies utilising this measure [35].

### Neck pain-related disability

The severity and impairment resulting from neck pain was assessed using the 10-item Neck Disability Index [36] (NDI). NDI items described neck pain severity and daily task impairments, such as reading and driving, as well as problems with concentration and headaches. Respondents indicated their pain and impairment levels on a 6-point response scale ranging from 0 to 5, with higher values indicating more severe symptoms. Vernon [37] recommends scoring the NDI out of 50 (the sum score), with disability categories of 0–4 = None, 5–14 = Mild, 15–24 = Moderate, 25–34 = Severe, and 34–50 = Complete. Internal consistency of NDI items was high (Cronbach’s α = 0.84).

### Health-related life quality

Health-related quality of life was measured using the 6-item European Quality of Life 5-Dimension 5-Level questionnaire [38] (EQ-5D-5 L). The EQ-5D-5 L measures the five mental and physical health dimensions of mobility, self-care, usual activities, pain/discomfort and anxiety/depression on the day of assessment. Response levels range from 1 to 5, with higher values indicating lower quality of life experiences. A sixth item enquires about the health level experienced on the day on a percentage scale, ranging from 0% (worst health) to 100% (best health). Responses to the EQ-5D-5 L were scored according to the Western preference pattern (WePP), which constitutes the recommended weighting for Western countries [39]. The highest score of the WePP index is 1, which indicates a state of complete physical and psychological health [40].

### Statistical analysis strategy

All analyses were conducted using IBM SPSS Statistics for Windows, version 29.0. To retain patient responses as much as possible, we allowed missing responses to psychometric measurements of up to 25% and mean imputed missing values on the item level (as opposed to the total score) to support unbiased regression model estimates [41]. If patients completed less than 75% of items on a specific scale measure, their responses were excluded from analysis.

We compared demographic characteristics and responses to first outpatient clinic assessment questionnaires between patient groups (employees without sickness absence, with graded sickness absence, or with full sickness absence) using Chi-square tests of independence (categorical variables) and One-way analysis of variance tests (continuous variables). General linear models for repeated measures were utilised to examine changes in neck and low back symptoms and health-related quality of life between completion of the baseline questionnaire and six months after. We conducted one test per outcome variable (low back pain-related disability, neck pain-related disability, and quality of life), adjusted for baseline health symptoms, and employed Bonferroni corrections for multiple comparisons. Based on the available literature on factors influencing health and sickness absence trajectories in patients with musculoskeletal disorders [9, 42, 43], we then reran the analysis with patient age, sex, education level, number of painful body regions, length of pain duration, and mental health symptoms at the time of the first assessment to observe whether the pattern of results remained.

## Results

### Sample characteristics

Table 1 presents baseline patient characteristics of employees with musculoskeletal disorders who returned the baseline and 6-months follow up questionnaires (*N* = 5143). A comparison of patients who returned the baseline questionnaire and those who returned both the baseline and 6-months follow-up questionnaires yielded significant differences in demographic characteristics. Specifically, those who completed both questionnaires were about 3 years older on average, were more commonly married or in a long-term partnership, and more commonly foreign nationals. There were, however, no significant differences on contracted work hours, sick leave status, or any of the outcome measures.

**Table 1:** Patient characteristics comparing patients who were on no, graded, or full sickness absence at time of initial consultation questionnaire (*N* = 5143)

	Full Sample (*N* = 5143)	No Sickness Absence (*n* = 2568)	Graded Sickness Absence (*n* = 1164)	Full Sickness Absence (*n* = 1411)	Test statistic *F*(*df*)/ χ^2^(*df*)
***Demographics***					
*Age*	44.70 (11.50)	44.52 (12.10)	45.08 (10.44)	44.70 (11.50)	0.93 (2)
*Sex*					82.08 (2)***
Female	2751 (53.5%)	1331 (51.8%)	752 (64.6%)	668 (47.3%)	
Male	2392 (46.5%)	1237 (48.2%)	412 (35.4%)	743 (52.7%)	
*Education Level*					188.29 (2)***
Primary, vocational, or senior high school	2730 (53.8%)	1150 (44.8%)	640 (55%)	940 (66.6%)	
College or university	2349 (46.2%)	1393 (54.2%)	513 (44.1%)	443 (31.4%)	
*Marital Status*					5.21 (2)
Married/In partnership	3872 (76.3%)	1954 (76.1%)	891 (76.5%)	1027 (72.8%)	
Single	1203 (23.7%)	585 (22.8%)	259 (22.3%)	359 (25.4%)	
*Nationality*					29.56 (2)***
Norwegian	4479 (87.1%)	2283 (88.9%)	1025 (88.1%)	1171 (83%)	
Other	664 (12.9%)	285 (11.1%)	139 (11.9%)	240 (17%)	
***Employment Characteristics***					
*Occupation (4 most common)*					422.9 (92)***
Health professional	507 (10.7%)	263 (10.2%)	134 (11.5%)	110 (7.8%)	
Sales worker	375 (7.9%)	177 (6.9%)	91 (7.8%)	107 (7.6%)	
Teaching professional	362 (7.6%)	199 (7.7%)	88 (7.6%)	75 (5.3%)	
Personal care worker	338 (7.1%)	138 (5.4%)	102 (8.8%)	98 (6.9%)	
*Current Work Ability Rating*	5.11 (2.96)	6.87 (2.07)	4.42 (2.04)	2.32 (2.64)	986.72 (2)***
*Physical and Mental Work Capability*					
Physical job demands	2.95 (1.18)	3.45 (0.96)	2.79 (1.03)	2.19 (1.21)	362.04 (2)***
Mental job demands	3.91 (1.03)	4.11 (0.86)	3.88 (1.02)	3.60 (1.22)	60.89 (2)***
*Job Satisfaction*	7.91 (2.09)	8.07 (1.92)	7.95 (2.00)	7.58 (2.42)	25.50 (2)***
*Perception of Employer’s RTW Motivation*	4283 (93.9%)	2015 (78.5%)	1089 (93.6%)	1179 (83.6%)	168.45 (2)***
***Pain Characteristics***					
*Duration of Current Pain*					148.73 (2)***
< 3 months	288 (%)	126 (%)	68 (%)	94 (%)	
3–12 months	1812 (%)	696 (%)	521 (%)	595 (%)	
≥ 1 year	2947 (%)	1694 (%)	560 (%)	693 (%)	
*Causes of Pain*					
Number reported	1.69 (1.36)	1.62 (1.37)	1.74 (1.30)	1.78 (1.37)	6.83 (2)***
*Number of Pain Areas*	6.28 (5.10)	5.73 (4.73)	6.71 (5.19)	6.92 (5.53)	30.71 (2)***
*Pain Experience*					
At rest	5.19 (2.25)	4.91 (2.26)	5.24 (2.16)	5.65 (2.24)	49.51 (2)***
During activity	6.16 (2.20)	5.65 (2.26)	6.28 (1.99)	6.99 (1.99)	179.75 (2)***
*Low Back Pain-related Disability (ODI)*	28.58 (13.74)	24.18 (11.89)	29.18 (11.97)	36.12 (14.87)	289.49 (2)***
*Neck Pain-related Disability (NDI)*	16.69 (7.51)	14.21 (6.65)	17.58 (6.54)	20.11 (8.13)	143.03 (2)***
***Treatment Characteristics***					
*Primary Diagnosis*					41.68 (14)***
* Neck*					
Non-specific condition	1054 (20.5%)	518 (20.2%)	251 (21.6%)	285 (20.2%)	
Neurol. dysfunction	129 (2.5%)	51 (2%)	38 (3.3%)	40 (2.8%)	
Other specific conditions	2 (0%)	0 (0%)	2 (0.2%)	0 (0%)	
* Back*					
Non-specific condition	2442 (47.5%)	1252 (48.8%)	509 (43.7%)	681 (48.3%)	
Neurol. dysfunction	452 (8.8%)	221 (8.6%)	91 (7.8%)	140 (9.9%)	
Other specific conditions	60 (1.2%)	40 (1.6%)	7 (0.6%)	13 (0.9%)	
Additional diagnosis	103 (2%)	59 (2.3%)	24 (2.1%)	20 (1.4%)	
Not given	901 (17.5%)	427 (16.6%)	242 (20.8%)	232 (16.4%)	
***General Health***					
*30-Day Number of Health Problems*	11.77 (5.66)	11.13 (5.59)	12.45 (5.57)	12.37 (5.74)	33.05 (2)***
*Health Related Quality of Life (EQ-5D-5L)*					
Health Today	55.89 (19.25)	62.24 (17.42)	53.01 (16.77)	46.19 (19.83)	307.45 (2)***
WePP Index	0.75 (0.16)	0.79 (0.13)	0.75 (0.14)	0.68 (0.19)	200.99 (2)***
***Mental Health Characteristics***					
*Fear-Avoidance Beliefs for Back Pain*					
Physical activity score	12.20 (5.69)	11.10 (5.56)	12.11 (5.31)	14.27 (5.66)	143.85 (2)***
Work score	19.86 (11.14)	14.45 (9.63)	22.14 (9.26)	27.85 (9.55)	900.88 (2)***
*Depression and Anxiety Symptoms*	1.91 (0.61)	1.81 (0.58)	1.96 (0.59)	2.07 (0.64)	89.32 (2)***

Note. * *p* < .05, ** *p* < .01, *** *p* < .001. Responses to survey items were not mandatory and, consequently, actual *N* and *n*s may vary due to missing data. Categorical variables are presented in *n*(%) and continuous variables are presented in *M*(*SD*). RTW is an acronym for return to work. ODI is the Oswestry Disability Index. NDI is the Neck Disability Index. EQ-5D-5 L is the European Quality of Life 5-Dimension 5-Level scale. WePP is the Western preference pattern of the EQ-5D-5 L

The final sample comprised of a greater number of women (*n* = 2751, 53.5%) than men (*n* = 2392, 46.5%) and had a mean age of 44.70 years (*SD* = 11.50). Clinician diagnostic information indicated that the majority of patients presented with non-specific back (*n* = 2442, 47.5%) and neck (*n* = 1054, 20.5%) conditions. Some patients presented with a primary diagnosis of neurological dysfunction of the neck or back (*n* = 581, 11.3%), and few with specific musculoskeletal disorders (*n* = 62, 1.2%). About half of patients were working without registered sick leave (*n* = 2568, 49.9%). The remainder of patients were on medically certified full sick leave (*n* = 1411, 27.4%) or followed a graded sick leave program (*n* = 1164, 22.6%, median sick leave % = 50, interquartile range = 10). All patient groups expressed positive employer beliefs, with those on graded sick leave agreeing most frequently with a statement that their employer would like to see them return to their workplace (*n* = 1089, 93.6%). A full list of occupations patients held is presented in the online supplementary material.

There were significant differences between sick leave groups across symptom characteristics at baseline, with most patterns indicating that patients on full sick leave reported the highest levels of low back- and neck pain-related disability, poor mental health and general health problems and patients without sick leave reported the lowest levels on these indicators. Those on graded sick leave consistently reported symptom levels between full and no sick leave groups. While those on full sick leave reporting the lowest levels of health-related quality of life, WePP scores across the sample fell below Norwegian population norms given by age and sex, which range from 0.83 (*SD* = 0.15) to 0.91 (*SD* = 0.10) [44]. Respondents who worked without sick leave reported the fewest psychological complaints, with mean scores below an identified cut-off score of 1.85 [45]. In contrast, those on graded and full sick leave reported psychological complaints above this threshold, indicating that those on graded and full sick leave were more likely to report symptoms of poor mental health.

### Changes in symptom severity

#### Low back pain-related disability

Table 2 shows the results of the general linear model for repeated measures analysis to compare the effect of patients’ sick leave status on low back pain-related disability at the time of completing a baseline questionnaire prior to their first outpatient clinic appointment (Time 1) and after 6 months (Time 2). There was a statistically significant effect of sickness absence group on ODI scores, *F*(2, 4972) = 309.76, *p* <.001, a significant effect of time on ODI scores, *F*(1, 4972) = 558.92, *p* <.001, and a significant time by group effect, *F*(2, 4972) = 46.75, *p* <.001, indicating that ODI symptom progression differed significantly between groups across time points.

**Table 2:** General linear model for repeated measures examining patients with low back pain-related disability who presented at a neck and back outpatient clinic and completed a questionnaire for the initial assessment (Time 1) and six months later (Time 2) (*N* = 4975). Higher scores indicate greater low back pain-related disability

Time 1			Time 2								
Model	M	SD	M	SD	M diff	SE diff	*p* diff	F	df	df(error)	*p*
*Mean values of low back pain-related disability*											
No sickness absence	24.15	11.91	21.74	13.09	-2.42	0.25	< 0.001				
Graded sickness absence	29.15	11.97	24.79	13.24	-4.36	0.37	< 0.001				
Full sickness absence	36.13	14.90	29.70	15.99	-6.43	0.34	< 0.001				
*Between subjects*											
Intercept								23971.63	1	4972	< 0.001
Sickness absence								309.76	2	4972	< 0.001
*Within subjects*											
Time								558.92	1	4972	< 0.001
Time * sickness absence								46.75	2	4972	< 0.001

**Fig. 2:**
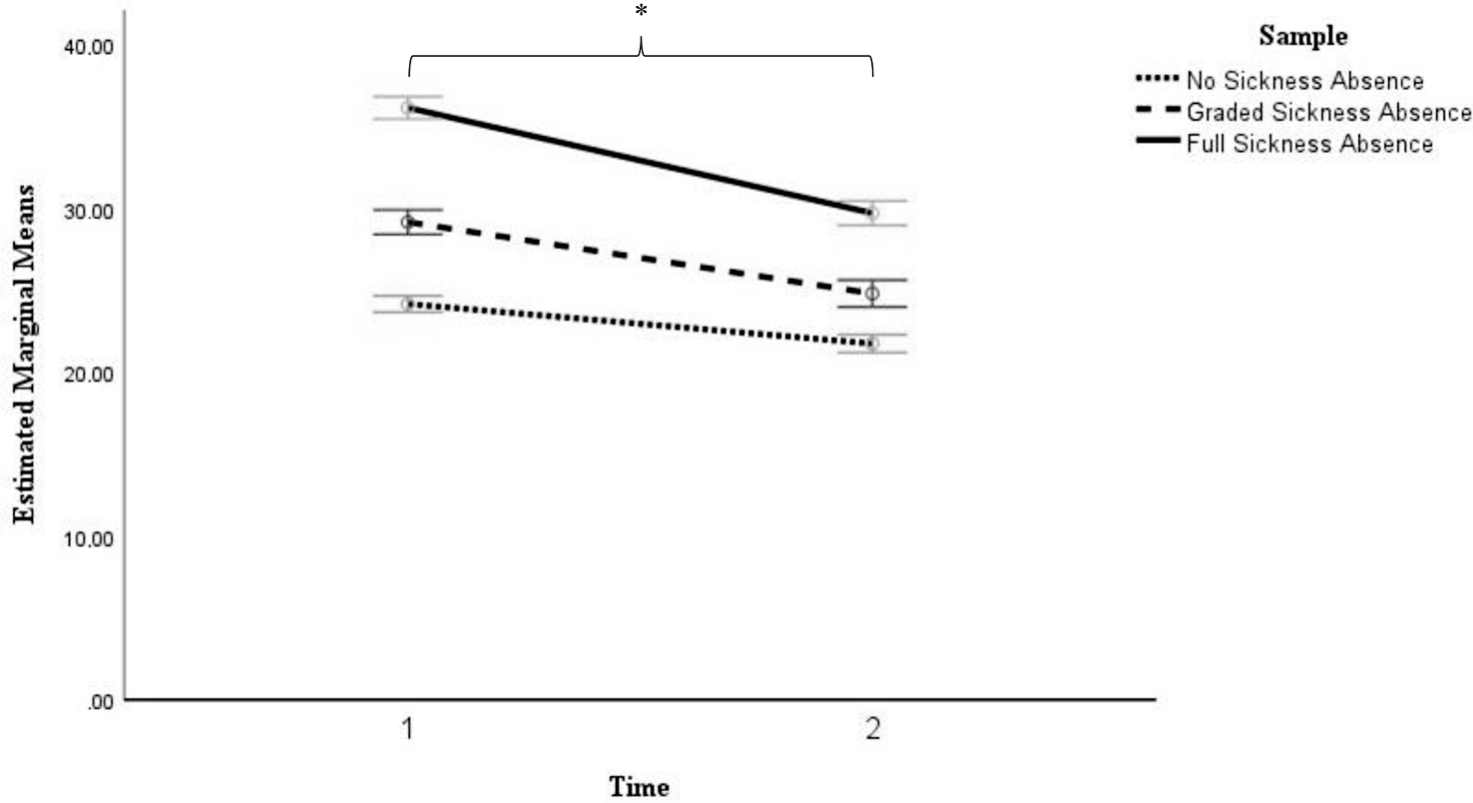
Illustration of the estimated marginal mean changes of low back pain-related disability between completing a questionnaire for the initial assessment (Time 1) and six months later (Time 2). *Note*. Error bars are the 95% Confidence Intervals; * denotes a significant effect of time

Figure 2 illustrates the mean symptom levels per group at Time 1 and Time 2. Patients prescribed full sickness absence consistently reported higher levels of back pain-related disability than those on graded sick leave. Patients who worked without sick leave reported the lowest levels of back pain-related disability at both questionnaire completion times with average scores in the lower moderate impairment range. ODI scores improved at Time 2 compared to Time 1 across groups. Although those on full sickness absence reported the greatest back pain-related disability improvements of approximately 6 points on average (*M* = -6.43, *SE* = 0.34), compared to those with graded (*M* = -4.36, *SE* = 0.37) and no sick leave (*M* = -2.42, *SE* = 0.25), they remained the group who observed the highest level of pain-related disability at 6 months.

The addition of covariates yielded significant effects for age (*F* (1, 4833) = 87.92, *p* <.001), education level (*F* (1, 4833) = 12.69, *p* <.001), number of painful body regions (*F* (1, 4833) = 67.41, *p* <.001), pain duration (*F* (1, 4833) = 35.71, *p* <.001), and mental health symptoms indicative of depression and anxiety (*F* (1, 4833) = 788.53, *p* <.001), however, the patterns of results pertaining to sickness absence (*F* (2, 4833) = 190.63, *p* <.001), time (*F* (1, 4833) = 45.84, *p* <.001), and the time by sickness absence interaction (*F* (2, 4833) = 34.06, *p* <.001) persisted and remained significant.

#### Neck pain-related disability

**Fig. 3:**
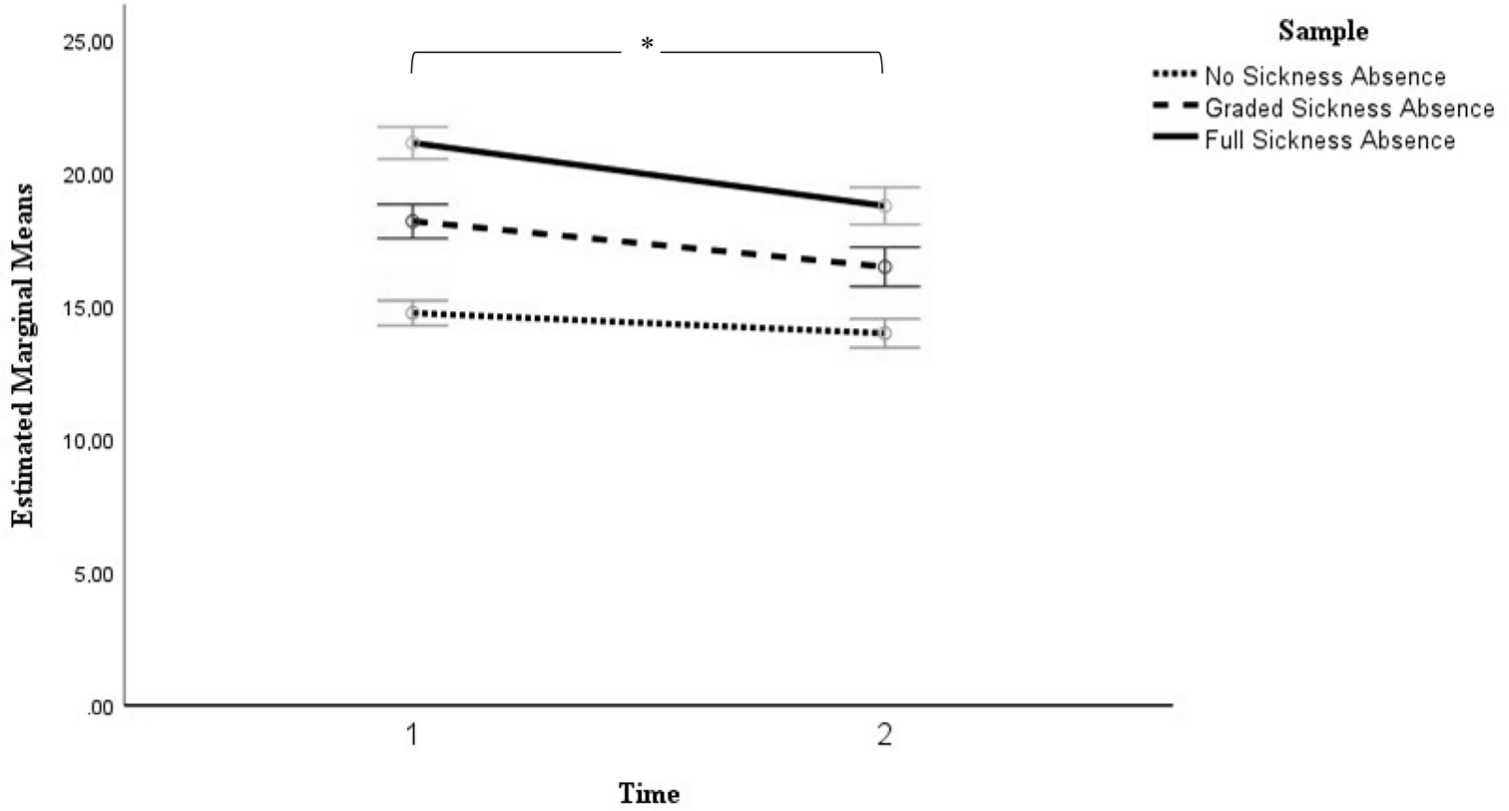
Illustration of the estimated marginal mean changes of neck pain-related disability between the initial assessment (Time 1) and six months later (Time 2). *Note*. Error bars are the 95% Confidence Intervals; * denotes a significant effect of time

Table 3 shows the results of the general linear model for repeated measures analysis to compare the effect of sick leave status on neck pain-related disability over six months. The main effects of sickness absence (*F* (2, 1764) = 110.99, *p* <.001) and time (*F* (1, 1764) = 106.27, *p* <.001) as well as the time by sickness absence interaction (*F* (2, 1764) = 10.50, *p* <.001) were significant. Patterns of results indicated that patients without sickness absence reported the lowest levels of neck pain-related disability across time points, whereby those on full sickness absence exhibited the highest pain-related disability levels (see also Fig. 3). Those on graded sick leave reported problems with neck pain-related disability between the other two groups with mean impairment reductions of 1.7 points (*SE* = 0.30) on the NDI scale across Times 1 and 2.

**Table 3:** General linear model for repeated measures examining patients with neck pain-related disability who presented at a neck and back outpatient clinic and completed a questionnaire for the initial assessment (Time 1) and six months later (Time 2) (*N* = 1767). Higher scores indicate greater neck pain-related disability

Time 1			Time 2								
Model	M	SD	M	SD	M diff	SE diff	*p* diff	F	df	df(error)	*p*
*Mean values of neck pain-related disability*											
No sickness absence	14.73	6.55	13.97	7.41	-0.76	0.22	< 0.001				
Graded sickness absence	18.18	6.21	16.46	7.32	-1.71	0.30	< 0.001				
Full sickness absence	21.12	7.86	18.75	9.19	-2.37	0.29	< 0.001				
*Between subjects*											
Intercept								10854.84	1	1764	< 0.001
Sickness absence								110.99	2	1764	< 0.001
*Within subjects*											
Time								106.27	1	1764	< 0.001
Time * sickness absence								10.50	2	1764	< 0.001

Adding covariates to the model yielded significant effects of patient age (*F* (1, 1703) = 17.49, *p* <.001), sex (*F* (1, 1703) = 9.18, *p* =.002), education level (*F* (1, 1703) = 4.68, *p* =.031), number of painful body regions (*F* (1, 1703) = 61.77, *p* <.001), current pain duration (*F* (1, 1703) = 19.39, *p* <.001), and depression and anxiety symptoms (*F* (1, 1703) = 339.34, *p* <.001) on neck pain-related disability. After the addition of covariates, the main effect of sickness absence and the time by sickness absence interaction remained significant (*p*s < 0.001), however, the main effect of time was not significant (*F* (1, 1703) = 3.49, *p* =.062).

#### Health-related life quality

**Fig. 4:**
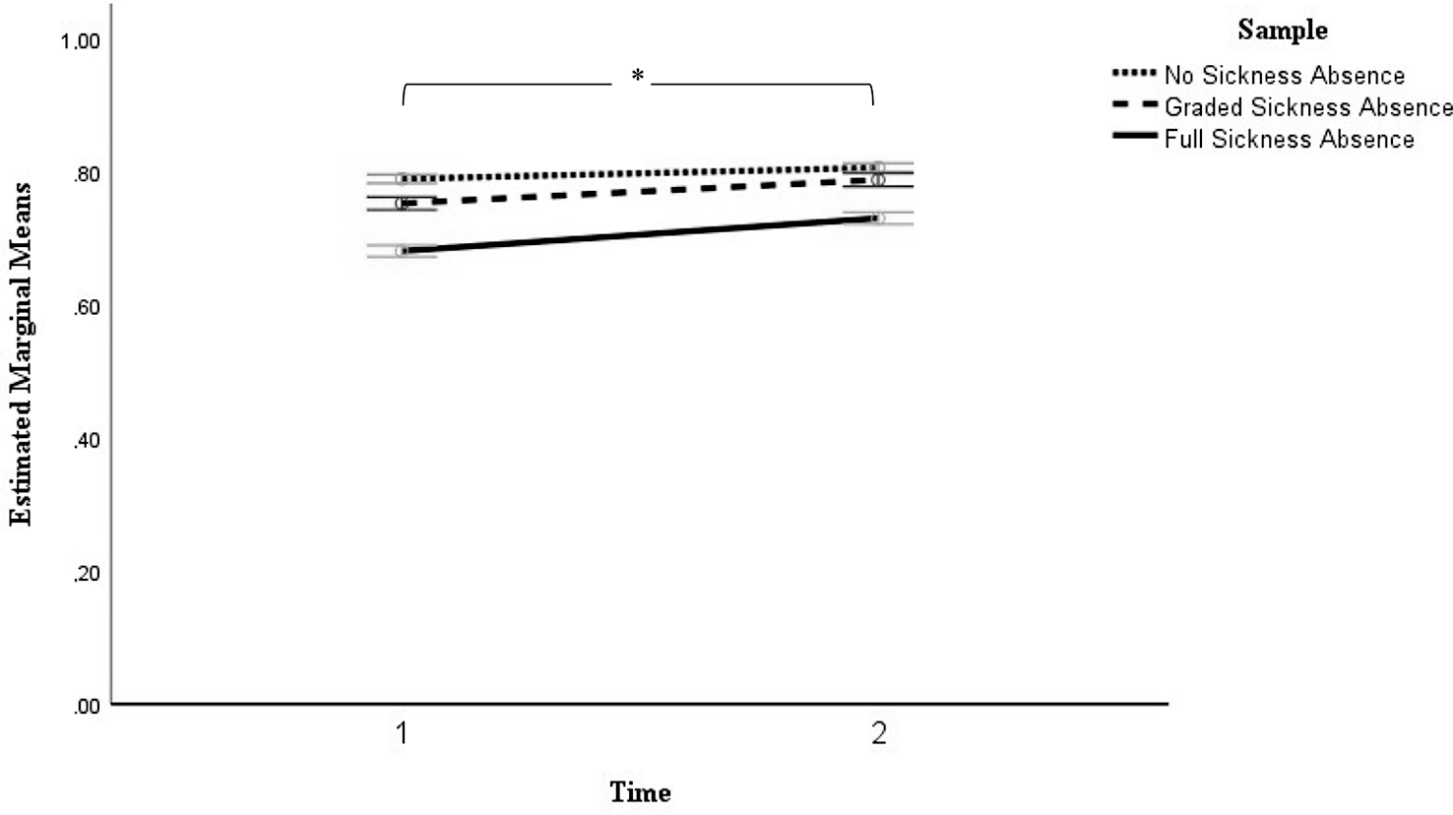
Illustration of the estimated marginal mean changes of perceived quality of life between the initial assessment (Time 1) and six months later (Time 2). *Note*. Error bars are the 95% Confidence Intervals; * denotes a significant effect of time

A general linear model for repeated measures analysis was conducted to observe changes in health-related quality of life between assessments at Times 1 and 2. Results are shown in Table 4 and mean changes are illustrated in Fig. 4. Main effects of sickness absence (*F* (2, 4429) = 175.75, *p* <.001) and time (*F* (1, 4429) = 186.31, *p* <.001) and the time by sickness absence interaction (*F* (2, 4429) = 17.84, *p* <.001) were significant. Patients without sickness absence reported the highest health-related quality of life across times, with the highest life quality outcomes 6 months after presenting at the outpatient clinic. Those on full sickness absence exhibited increases in health perceptions 6 months after the first outpatient clinic assessment but remained the group with the lowest health experiences. Those on graded sick leave experienced health-related quality of life levels between the no and full sickness groups.

**Table 4:** General linear model for repeated measures examining Health-related quality of life experiences of patients who presented at a neck and back outpatient clinic and completed a questionnaire for the initial assessment (Time 1) and six months later (Time 2) (*N* = 4432). Higher scores indicate greater perceived quality of life

Time 1			Time 2								
Model	M	SD	M	SD	M diff	SE diff	*p* diff	F	df	df(error)	*p*
*Mean values of the* Western preference pattern											
No sickness absence	0.79	0.13	0.81	0.14	0.02	0.00	< 0.001				
Graded sickness absence	0.75	0.14	0.79	0.15	0.04	0.01	< 0.001				
Full sickness absence	0.68	0.20	0.73	0.20	0.05	0.00	< 0.001				
*Between subjects*											
Intercept								119434.44	1	4429	< 0.001
Sickness absence								175.75	2	4429	< 0.001
*Within subjects*											
Time								186.31	1	4429	< 0.001
Time * sickness absence								17.84	2	4429	< 0.001

After adding covariates to the model, sex (*F* (1, 4310) = 14.96, *p* <.001), painful body regions (*F* (1, 4310) = 65.88, *p* <.001), current pain duration (*F* (1, 4310) = 4.70, *p* =.030), and depression and anxiety symptoms (*F* (1, 4310) = 841.71, *p* <.001) were significant. The main effects of time and sickness absence and the interaction effect of time and sickness absence remained significant after the inclusion of covariates.

## Discussion

This article investigated changes in neck and back pain-related disability and quality of life experiences of patients with and without medically certified sick leave who were referred to specialised outpatient clinics for musculoskeletal complaints.

We firstly examined whether patients on full, graded, or without sick leave differed in their demographic, health, mental health, treatment, and employment characteristics. Descriptive results indicated groups differed across most dimensions in small but consistent ways, indicating that those on full sickness absence had the lowest health and quality of life experiences compared to those on graded and no sickness absence, respectively. This could be due to doctors prescribing sick leave more often to patients experiencing more severe problems, including comorbid mental health conditions [46], or be indicative of a protective effect of work [47–49]. All groups reported quality of life experiences below population norms [44]. This was expected given patients’ reduced health status around the time of admittance.

Patients on graded sick leave generally reported neck and low back pain-related disability in the moderate ranges, with scores generally falling between the full sickness absence and no sickness absence groups. This supports the understanding that patients who are prescribed graded sickness absence are unable to work full time but remain capable of working in a reduced capacity. Interestingly, patients on graded sick leave expressed the highest agreement with a statement that their employers would like to see them back at work, suggesting that a well organised graded sick leave and work arrangement may foster positive workplace beliefs and relationships [50].

We further examined pain-related disability and health-related life quality changes between the time of completing a baseline questionnaire in preparation of the first visit at a neck and back pain outpatient clinic and six months later, comparing patients based on their prescribed sick leave status. Generally, patterns of results indicated that pain-related disability and life quality improved across groups over six months. This could be explained by the fact that patients were referred to specialist healthcare services when their impairment was particularly concerning. Six-months symptom improvements may thus support the therapeutic value of specialised care, although this was not examined in the current study. Results could also signify an effect of remission of symptoms over time. Particularly patients with higher pain-related disability at baseline, like the full sickness absence group, could have experienced symptom reductions due to regression towards the mean patterns common in observational data [51]. Despite the overall improvements noted, findings were also indicative of the persistent nature of musculoskeletal disorders. For example, reported neck pain-related disability improvements in each of the sickness absence groups, while significant, remained below minimal clinically important difference values [52]. Additionally, when previously identified covariates of health and sickness absence changes in patients with musculoskeletal disorders were accounted for [9, 42, 43], 6-months neck pain-related disability improvements across groups did not hold.

Patients with medical certificates for full sick leave experienced the greatest reductions in pain-related disability across time compared to the other groups, however, full sickness absence patients consistently experienced higher neck and low back pain-related disability as well as lower perceived life quality across groups. Lower back pain-related disability of patients prescribed full sickness absence constituted a significant and clinically meaningful change under a 5-point change cut off [for a discussion on the applicability of single point estimates, see [53]]. Patients on graded sick leave or without sick leave did not note clinically meaningful changes in lower back pain-related disability. It is possible that those who experienced the highest initial pain-related disability levels had more potential for improvement compared to those who had lower pain-related disability levels to begin with.

Our aim was to explore whether sick leave status was indeed indicative of patients’ health status, as it is often implied in the return-to-work literature, despite known psychosocial influences of incapacity in unspecified musculoskeletal disorders [54] and the possibility of sick workers exhibiting presenteeism, which may delay alleviation of pain-related disability [24, 55]. Overall, results indicated that the level of prescribed sickness absence corresponded to patients’ self-reported severity of health problems present. This finding is consistent with Standal and colleagues’ [20] findings suggesting that self-reported health was lower in Norwegians who were prescribed full sick leave compared to those prescribed graded sick leave. Other research groups have also linked health experiences to sick leave patterns. For example, Rysstad and colleagues [56] found that higher self-perceived health was associated with higher odds of belonging to the fast (versus slowed) decreasing sickness absence trajectory group [56]. Changes in health perceptions and the type of sick leave, however, were not integrated into the trajectory analysis, making it difficult to determine whether different approaches to balancing sick leave with work had any effect. Our findings support the notion that patients’ pain and functional impairment perceptions correlate with practitioners’ decisions to prescribe full, graded, or no sick leave.

This study has several strengths and limitations. This research constitutes an early examination of neck and low back pain patient and clinician information collected by the NNRR. The combination of self-report patient symptom data with objective NAV employment information, which is used to allocate payments to recipients, constitutes a significant strength of this research. Drawing from government records increased the accuracy of the data utilised for analysis. In support of validity considerations, the naturalistic setting in which patient information was collected constitutes another strength of this research. However, relating to these strengths, several limitations need to be noted. First, due to the naturalistic nature of the assessed data, complete records could not be retrieved in 9086 cases, which resulted in a large number of exclusions due to missing questionnaire data and insufficient information to determine eligibility. Consequently, the present sample is restricted to patients who were referred to outpatient clinics, consented to be included in research, and provided sufficient data to be included in the present analysis. The present findings do not represent all Norwegian patient groups with musculoskeletal conditions. Second, those with more severe and chronic conditions may have been referred to, and sought, additional treatment avenues. Therefore, 6-months changes in pain-related disability levels cannot be solely attributed to patients’ sickness absence status. Similarly, additional influencing factors such as patients’ workplace environments, psychosocial conditions, and attitudes toward return to work were not assessed [57–59]. Finally, sickness absence status was determined based on the first appointment at an outpatient clinic for neck and back pain problems. This did not consider employment or sick leave changes during the six months following outpatient clinic intake. Thus, group allocation improved for 1705 (33.15%) and worsened for 461 (8.96%) patients at the second assessment time point. However, for the purposes of this research question, i.e., to determine whether health changes differ across groups after the initial assessment, we retained the original group allocations made at the first visit to an outpatient clinic. It also needs to be noted that only patients were selected for the present analysis who had work contracts of 30 h or more per week. The reason for this decision was to draw clear distinctions between sickness absence groups, whereby patients allocated to the graded sick leave group would indeed work less hours on average than those allocated to the no sick leave group. We acknowledge that this is an artificially drawn distinction to aid in the interpretation of group allocation and results and does not accurately reflect real-world complexities in which employees have part-time work contracts of 30 h or less per week. For this reason, and due to the analysis utilising data derived from the Norwegian healthcare and welfare systems, the present study lacks generalisability to other settings and countries.

Future studies should examine the relation between neck and back pain-related disability, life quality and sick leave status among patients with musculoskeletal disorders in purpose-designed studies with rigorous study designs, including randomised controlled trials. Future research should further demonstrate the boundary conditions under which graded, full, and no sick leave entitlements are most or least associated with favourable health and return-to-work outcomes.

## Conclusions

First examinations using data derived from a novel neck and back pain registry demonstrate that a health gain hypothesis of graded sick leave in patients with musculoskeletal disorders was not supported, nor was the notion supported that partial work participation may prolong ill-health through presenteeism. Instead, each examined sickness absence status group reported significant pain-related disability reductions over time. This is important to demonstrate because the usefulness of work participation should be reduced if it hindered functional recovery from musculoskeletal disorders. Self-reported pain-related disability and life quality changes further suggest that those on full sickness absence continue to exhibit more severe pain-related disability, despite higher mean improvements, and lower health-related quality of life after six months compared with patient groups who engage in work activities prior to their initial outpatient clinic appointment. The full sickness absence musculoskeletal patient group appears to subsume clinical presentations consistent with more severe pain that is more difficult to treat and shows less favourable prognosis. Whether these findings support the importance of early detection and timely referral to specialised outpatient clinics is subject to further investigation under controlled conditions.

## Electronic supplementary material

Below is the link to the electronic supplementary material.

Supplementary Material 1

## Data Availability

The datasets generated and analysed during the current study are not publicly available due to access requiring approval from the Norwegian Labour and Welfare Administration and the Norwegian Neck and Back Register council but are available from the corresponding author on reasonable request.

## Footnotes

1 In the remainder of this article, we will use the phrase *outpatient clinics* when referring to neck and back pain outpatient clinics in the specialist health sector.

2 Sickness absence allocations between 0 and 20% and 95–100% do not exist in the Norwegian health and welfare system and are therefore not considered in this article.
