# Supplementary material for: Health consequences of graded, full, and no sickness absence among workers with musculoskeletal disorders: health profiles and six-months symptom changes of patients referred to Norwegian outpatient clinics for chronic neck and back pain
Source: BMC Musculoskelet Disord. 2025 May 1;26:432. doi: 10.1186/s12891-025-08570-7 (PMC12044821; doi:10.1186/s12891-025-08570-7)
Supplement: Supplementary file 1 — Supplementary Material 1 [file 12891_2025_8570_MOESM1_ESM.docx]

**Supplementary Material**

Professions provided by patients in a baseline questionnaire completed in preparation of a first visit to a Norwegian neck and back pain outpatient clinic.

| Occupation^a^ | Frequency | Percent |
| --- | --- | --- |
| Health professionals | 507 | 9,9 |
| Sales workers | 375 | 7,3 |
| Teaching professionals | 362 | 7,0 |
| Personal care workers | 338 | 6,6 |
| Personal service workers | 327 | 6,4 |
| Building and related trades workers, excluding electricians | 325 | 6,3 |
| Business and administrative professionals | 272 | 5,3 |
| Drivers and mobile plant operators | 226 | 4,4 |
| Science and engineering professionals | 190 | 3,7 |
| General and keyboard clerks | 190 | 3,7 |
| Health associate professionals | 184 | 3,6 |
| Science and engineering associate professionals | 124 | 2,4 |
| Administrative and commercial managers | 108 | 2,1 |
| Metal, machinery and related trades workers | 105 | 2,0 |
| IT professionals | 103 | 2,0 |
| Legal, social and cultural professionals | 100 | 1,9 |
| Business and administration associate professionals | 100 | 1,9 |
| Stationary plant and machine operators | 92 | 1,8 |
| Cleaners and helpers | 89 | 1,7 |
| Legal, social, cultural and related associate professionals | 76 | 1,5 |
| Protective services workers | 75 | 1,5 |
| Electrical and electronic trades workers | 69 | 1,3 |
| Customer services clerks | 39 | 0,8 |
| Assemblers | 35 | 0,7 |
| Production and specialized service managers | 30 | 0,6 |
| Subsistence farmers, fishers, hunters and gatherers | 27 | 0,5 |
| Chief executives | 18 | 0,3 |
| Handicraft and printing workers | 13 | 0,3 |
| Numerical and material recording clerks | 11 | 0,2 |
| Armed forces occupations, other ranks | 9 | 0,2 |
| Commissioned armed forces officers | 8 | 0,2 |
| Information and communications technicians | 7 | 0,1 |
| Refuse workers and other elementary workers | 5 | 0,1 |
| Market-oriented skilled agricultural workers | 4 | 0,1 |
| Market-oriented skilled forestry, fishery and hunting workers | 4 | 0,1 |
| Other clerical support workers | 2 | 0 |
| Food processing, wood working, garment and other craft and related trades workers | 2 | 0 |
| Labourers in mining, construction, manufacturing and transport | 2 | 0 |
| Food preparation assistants | 2 | 0 |
| Hospitality, retail and other services | 1 | 0 |
| Agricultural, forestry and fishery labourers | 1 | 0 |

*Note*. ^a^Self-report occupations in the Norwegian language were translated into English and categorised following the International Standard Classification of Occupations (ISCO). Responses to the occupational item were optional (missing *n* = 392) and a further 194 statements could not be translated or coded.
